# The BMP inhibitor DAND5 in serum predicts poor survival in breast cancer

**DOI:** 10.18632/oncotarget.7498

**Published:** 2016-02-19

**Authors:** Yayun Chi, Ling Yao, Xin Hu, Sheng Huang, Naisi Huang, Shan Li, Zhiming Shao, Jiong Wu

**Affiliations:** ^1^ Department of Breast Surgery, Breast Cancer Institute, Shanghai Cancer Center, Department of Oncology, Shanghai Medical College, Fudan University, Shanghai, 200032, China; ^2^ Department of Breast Surgery, Breast Cancer Institute, Shanghai Cancer Center, Collaborative Innovation Center of Cancer Medicine, Department of Oncology, Shanghai Medical College, Fudan University, Shanghai 200032, China

**Keywords:** DAND5, breast cancer, biomarker, secreted factor, prognosis

## Abstract

**Background & Aims:**

Breast cancer (BC) is prevalent worldwide malignant cancer. Improvements in timely and effective diagnosis and prediction are needed. As reported, secreted DAND5 is contributed to BC metastasis. We aim to assess whether DAND5 in peripheral blood serum could determine BC-specific mortality.

**Methods:**

We used immunohistochemistry staining to detect DAND5 expression in our BC tissue array including 250 samples. Angiogenesis assay and xenograft mice model were used to examine the secreted DAND5 function in BC progression. Serum concentration of DAND5 was examined by ELISA in 1730 BC patients. Kaplan-Meier and adjusted Cox proportional hazards models were utilized to analyze the prognosis and survival of BC patients.

**Results:**

Tissue array results showed that positive DAND5 staining cases displayed a higher likelihood of occurrence of disease events (HR=5.494; 95% CI: 1.008-2.353; P=0.048) in univariate analysis and remained the same trend in multivariate analysis (HR=2.537; 95% CI: 1.056-6.096; P=0.037). DAND5 positive patients exerted generally poor DFS (P=0.041) in the Kaplan-Meier survival analysis. Furthermore, secreted DAND5 promoted tumor growth and angiogenesis in vitro and in vivo. In addition, positive DAND5 in BC patients serum was associated with increased risk of disease events occurrence (univariate: HR=1.58; 95% CI: 1.206-2.070; P=0.001; multivariate: HR=1.4; 95% CI: 1.003-1.954; P=0.048) in univariate and multivariate survival analysis. In the Kaplan-Meier analysis, serum DAND5 positively correlated with poor DFS (P=0.001) and DDFS (P=0.002).

**Conclusions:**

DAND5 was correlated with poor survival and could serve as an easily detectable serum biomarker to predict the survival of breast cancer.

## INTRODUCTION

Breast cancer is the most common malignancy among women and represents an important worldwide public health issue [1, 2]. It is the major cause of cancer-related death in women and treatment is particularly difficult in patients with tumor metastasis [3]. Despite recent improvements in survival rates, many patients relapse and the majority of these patients die from disseminated metastatic disease [4], [5]. Therefore, there is a disquieting need for the identification of diagnosing markers which can be able to diagnose in early stage of breast carcinogenesis and progression.

Some breast cancer markers are already used in the clinic, such as tissue markers (hormone receptors, human epidermal growth factor-2, urokinase plasminogen activator, plasminogen activator inhibitor, p53 and cathepsin D) and genetic markers (BRAC1 and 2 and gene expression microarray technique, etc.) [4, 6, 7]. Additionally, due to the appropriate price and convenient detection, serum markers are widely used in the early screening test. The serum makers (CA 15-3 [8], BR 27-29 [9], MCA, CA 549, carcino embryonic antigen, oncoproteins, and cytokeratins [10]) are used in present diagnosis [11]. Many other secreted factors are used in other tumors, including AFP (α-fetoprotein), CEA (carcinoembryonic antigen) and PSA (prostate-specific antigen). They are all secreted by cancer cells through cellular membranes or exosomes and can be easily detected in the patients' body fluid.

DAND5, a secreted antagonist of TGF- β ligands was reported to be critical for BC metastasis [12]. The aims of the present study were to examine the serum DAND5 concentration in BC patients' peripheral blood and to examine the relationship between DAND5 and BC patients' prognosis. We tried to find novel serum biomarkers for BC metastasis and to promptly predict the metastasis and validate their prognosis through conveniently monitoring the biomarkers.

## RESULTS

### DAND5 overexpression directly associated with poor disease-free survival

**Figure 1:**
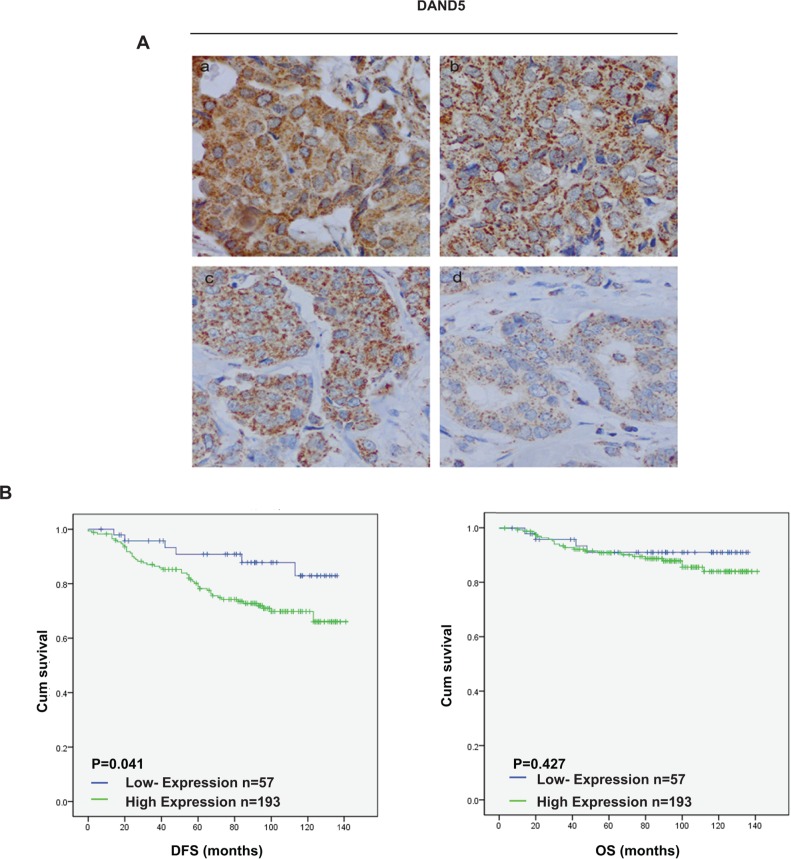
DAND5 expression profile in breast cancer tissue microarrays **A.** DAND5 immunostaining was determined in breast cancer and scored as (1) (2) low expression, (3) (4) high expression. All immunohistochemical photomicrographs are magnified 400×. **B.** Relationship between DAND5 expression and disease free survival (DFS)/overall survival (OS). *P* values were calculated using the unadjusted log-rank test.

As reported, antagonist of TGF-β ligands DAND5 could induce dormant breast cancer cells to undergo reactivation in the lung. To further validate this result and examine its relationship with breast cancer prognosis, tissue microarrays including 250 BC patients were immunostained for DAND5 (representative images, positive: a-c, negative: d, Figure 1A). Positive staining of DAND5 was detected in 77.2% (n=193; 77.2% positive, 22.8% negative) of tumors according to the scoring criterion described in the method. DAND5 mainly localized in cytoplasm and displayed a granular staining in accordance with its role as a secreted protein. We further analyzed the relationship between clinicopathologic features and DAND5 expression level. It showed that DAND5 had a negative relationship with ER status (P=0.044) and patients age (P=0.045) (Table 1). There was no correlation between the DAND5 level and menopausal status (P=0.052), tumor size (P=0.769), node status (P=0.308), HER2 status (P=0.074), differentiation (P=0.923) and TNM stage (P=0.535).

**Table 1:** Relationship between DAND5 expression and clinicopathological features in 250 breast cancer patients for IHC detection

Characteristics	DAND5		Number of patients (%)	P^a^ value
	Low n (%)	High n (%)		
**total**	**57(22.8)**	**193(78.2)**	**250**	
Age (mean 51.9, SD 9.524, median 51, range 29-85)				**0.045**
<50	34(13.6)	86(34.4)	120(48)	
≥50	23(9.2)	107(42.8)	130(52)	
Menopausal status				0.052
Pre	31(12.4)	77(30.8)	108(43.2)	
Post	26(10.4)	116(46.4)	142(56.8)	
Tumor size (cm)				0.769
2cm	25(10)	90(36)	115(46)	
>2,5cm	28(11.2)	92(36.8)	120(48)	
>5cm	4(1.6)	8(3.2)	12(4.8)	
Node status				0.308
Negative	38(15.2)	113(45.2)	151(60.4)	
Positive	19(7.6)	78(31.2)	97(38.8)	
ER status				**0.044**
Negative	40(16)	110(44)	150(60)	
Positive	17(6.8)	83(33.2)	100(40)	
HER-2 status				0.074
Negative	34(13.6)	114(45.6)	148(59.2)	
Positive	23(9.2)	78(31.2)	100(40)	
Differentiation				0.923
I	1(0.4)	1(0.4)	2(0.8)	
II	41(16.4)	143(57.2)	184(73.6)	
III	15(6)	46(18.4)	61(24.4)	
TNM stage				0.535
I	17(6.8)	57(22.8)	74(29.6)	
II	33(13.2)	99(39.6)	132(52.8)	
III	7(2.8)	35(14)	42(16.8)	

Abbreviations: ER, estrogen receptor; HER-2, human epidermal growth factor receptor 2

a Based onPearson χ2 test except for surgery type and radiation therapy, for which P is based on Fisher's exact test.

To validate the clinical significance of DAND5 overexpression in breast cancer, we analyzed the relationship between DAND5 expression and patient disease-free survival. Both univariate and adjusted multivariate survival analysis revealed statistical significant difference in DAND5 positive and negative group. In this cohort, positive DAND5 staining cases showed a higher likelihood of occurrence of disease events (HR=5.494; 95% CI: 1.008-2.353; P=0.048, Table 2) in univariate analysis and remained the same trend in multivariate analysis (HR=2.537; 95% CI: 1.056-6.096; P=0.037, Table 3). Besides, large tumor size also showed a higher likelihood of occurrence of disease events (HR=2.708; 95% CI: 1.086-1.715; P=0.021, Table 2) in univariate analysis and remained the same trend in multivariate analysis (HR=1.806; 95% CI: 1.085-3.005; P=0.023, Table 3). Additionally, DAND5 positive patients exerted generally poor DFS (P=0.041) in the Kaplan-Meier survival analysis (Figure 1B). The OS showed the same trend although it had no statistical significance (P=0.47). Furthermore, DAND5 expression was much higher in breast cancer tissues than in normal tissues at both mRNA level and protein level (Supplementary Figure S1). As recent study identified that DAND5 might have important roles in waking dormant breast cancer stem cells in the lung, we further analyzed the data and found that there were 17 patients in 250 tissue array group who finally developed lung metastasis. Only one patient displayed DAND5 negative. All the other 16 patients were turned to be DAND5 positive. These results strongly indicated that DAND5 expression directly associated with recurrent diseases of patients with breast cancer.

**Table 2:** Univariate regression model of prognostic covariates in 250 BC patients

Variable	HR	95.0% CI		*p* value
		Lower	Upper	
Age(<50/>=50)	0.815	0.553	1.595	0.939
Menopausal status(negative/positive)	0.1	0.914	2.78	1.594
ER(negative/positive)	0.388	0.456	1.356	0.787
PR(negative/positive)	0.027	0.185	0.903	0.409
TNM(I, II, III)	0.003	1.24	2.767	1.852
Pathological stage(I, II, III)	0.035	1.041	2.98	1.761
Her2 status(negative/positive)	0.809	0.548	1.6	0.936
Node status(negative/positive)	0.003	1.304	3.819	2.232
Tumor size (2 cm, >2,5cm, >5 cm)	2.708	1.086	1.715	**0.021**
DAND5 (negative/positive)	5.494	1.008	2.353	**0.048**

Abbreviations: CI, confidence interval; ER status, estrogen receptor status; PR status, prostrogen receptor status;HER-2, human epidermal growth factor receptor 2; HR, hazard ratio.

**Table 3:** Multivariate regression model of prognostic covariates in 250 BC patients

Variable	HR	95.0% CI		*p* value
		Lower	Upper	
Age(<50/>=50)	0.573	0.284	1.155	0.12
Menopausal status(negative/positive)	2.022	0.971	4.211	0.06
ER(negative/positive)	1.179	0.561	2.479	0.664
PR(negative/positive)	0.367	0.137	0.98	**0.046**
TNM(I, II, III)	1.029	0.552	1.916	0.929
Pathological stage(I, II, III)	1.594	0.887	2.865	0.119
Her2 status(negative/positive)	0.874	0.481	1.586	0.657
Node status(negative/positive)	1.918	0.924	3.98	0.081
Tumor size (2cm,>2,5cm,>5cm)	1.806	1.085	3.005	**0.023**
DAND5 (negative/positive)	2.537	1.056	6.096	**0.037**

Abbreviations: CI, confidence interval; ER status, estrogen receptor status; PR status, prostrogen receptor status;HER-2, human epidermal growth factor receptor 2; HR, hazard ratio.

### DAND5 promoted tumor growth and angiogenesis *in vitro* and *in vivo*

**Figure 2:**
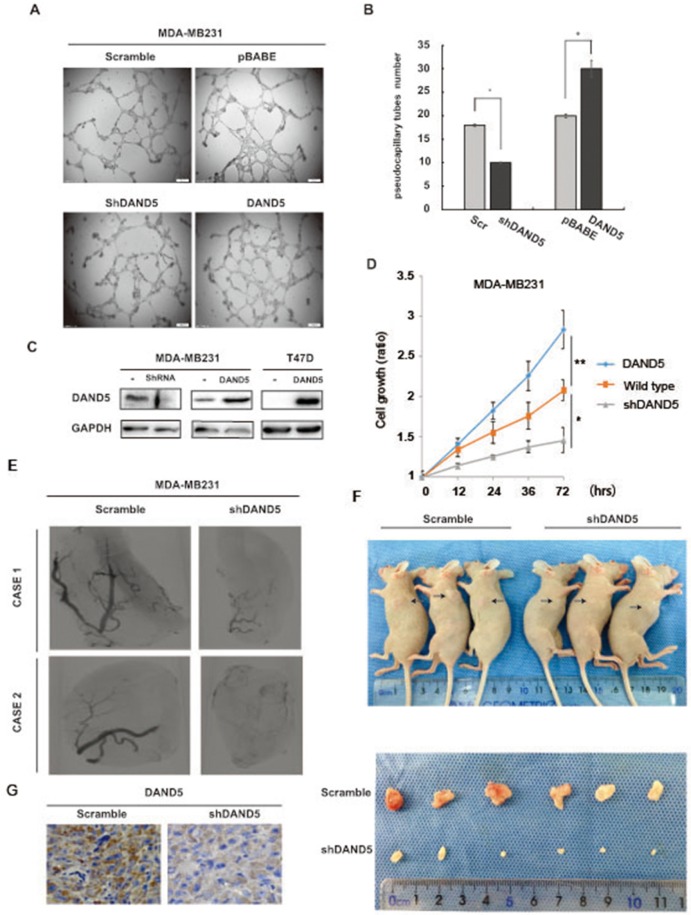
DAND5 promoted tumor proliferation and angiogenesis *in vitro* and *in vivo* **A.** Representative pictures of pseudocapillary formation in matrigel from HUVECs in 0.1% FBS exposed to breast cancer cell culture at 12 h after cell seeding. **B.** Quantification of pseudocapillaries obtained by counting numbers of complete circles/wells. Numbers represent the mean of 6 samples ± SEM of three experiments run in triplicate. **C.** MDA-MB231 DAND5 and knock down stable cell lysates and T47D DAND5 stable cell lysates were immunoblotted using DAND5 antibody. **D.** MDA-MB231 wild type, DAND5 and knock down stable cells were seeded in 96 well plate and cultured for the times as indicated, the cell growth was analyzed with CCK-8 assay. *P <0.05. **P<0.001. **E.** Inhibition of DAND5 by shRNA inhibited the vascularization of tumors in mice. The images were reconstructed using the filtered back projection (FBP) algorithm. Tumorigenesis was obtained after injection of MDA-MB231 cells or MDA-MB231 shDAND5 cells. **F.** Image of representative tumors from MDA-MB231 or MDA-MB231 shDAND5 xenografts harvested at end point. **G.** Images to visualize positive staining of DAND5 in xenograft tumor harvested at end point. Bars: 20 um, magnification × 400.

As DAND5 is a secreted BMP inhibitor, we speculated that the secreted DAND5 might affect the capillary network from the surrounding host tissue, which is needed both in cancer proliferation and in cancer metastasis [13]. To test our hypothesis, we constructed the MDA-MB231 stable cell lines with DAND5 overexpression or DAND5 knock down. DAND5 knock down efficiency and over-expression level were confirmed by Western blot in Figure 2C. Using ELISA assay, we first detected the concentration of the secreted DAND5 in cell cultures. The average concentration of MDA-MB231 DAND5 overexpression stable cell culture was 12.87 ng/ml, the wild type MDA-MB231 cell line was 2.02 ng/ml and the DAND5 knocked down cell line was undetectable. HUVEC cells were then treated with these different culture mediums. 48 hours later, angiogenesis was assessed. The data showed HUVECs were organized in a network of pseudocapillary tubes with the DAND5 cell cultures (Figure 2A). DAND5 increased the number of pseudocapillaries in terms of completed circles, while shDAND5 decreased the numbers (Figure 2B for quantification). These data suggest that DAND5 promoted pseudocapillary formation *in vitro*.

In order to examine whether this function was related with TGFβ signaling, TGF blocker was used for further examination. However, when TGFβ blocker Pirfenidone was added, angiogenesis was inhibited. Meanwhile, Pirfenidone inhibited angiogenesis induced by DAND5 (Supplementary Figure S2). As reported, BMPs function was varied in different isoforms and cancers. For example, BMP-2, BMP-4, BMP-6 and BMP-7 are pro-angiogenic factors, while BMP-9 and BMP-10 are anti-angiogenic factors [14]. We speculated that as antagonist of TGFβ ligands and BMP signaling blocker, DAND5 finally induced angiogenensis. The exact mechanism needs further investigation.

To further examine whether DAND5 was related to the cell growth, we carried out the cell proliferation assay with MDA-MB231 cells. The data showed DAND5 knock down MDA-MB231 cells grew slowly compared to the control.

The function of DAND5 was then examined in xenograft mice models. MDA-MB231 scramble cells or MDA-MB231 shDAND5 stable cells were injected into the mice mammary pat to form the xenograft tumors. After 2 weeks, the DAND5 concentration in mice serum in two groups was examined. The average serum concentration of scramble group is 0.29 ng/ml, while shDAND5 groups were undetectable. As shown in Figure 2E and 2F, both tumor size and the MVD (micro-vascular density) were decreased by shDAND5 in the MDA-MB231 shDAND5 group compared to the control groups. DAND5 expression in xenografts was also detected by IHC staining. As shown in Figure 2G, DAND5 expression was significantly decreased in shDAND5 group compared with control group. Based on these results, we speculated that DAND5 could promote the breast cancer cell growth and induce angiogenesis in breast cancer. The serum DAND5 might be more sensitive and easy to detect and possible to serve as a suitable serum biomarker to predict the prognosis of breast cancer patients.

### Serum DAND5 in breast cancer patients linked to opposing outcomes by ELISA detection

**Figure 3:**
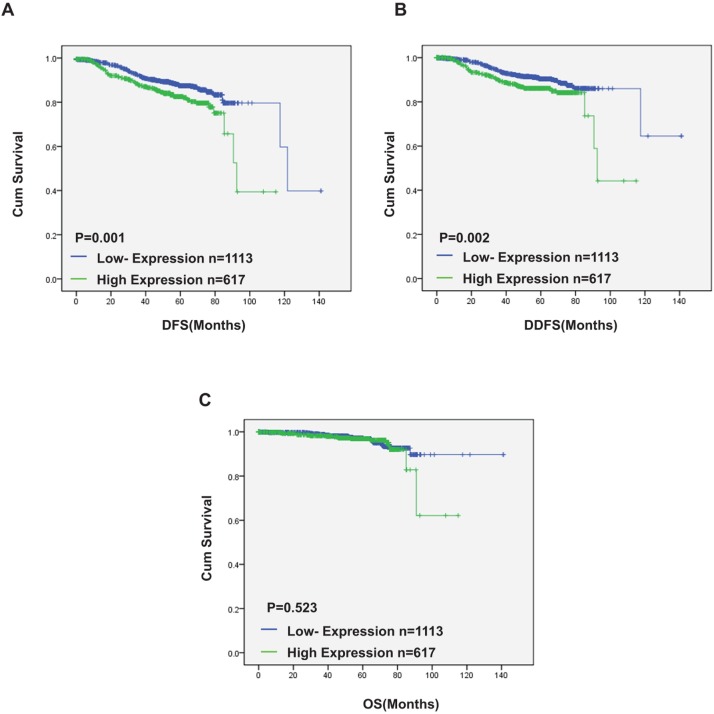
Serum DAND5 in breast cancer patients linked to opposing outcomes by ELISA detection Relationship between DAND5 expression and disease free survival (DFS)/overall survival (OS). *P* values were calculated using the unadjusted log-rank test.

To test our hypothesis and evaluate the predictive prognostic significance of DNA5 in serum, we performed ELISA detection in peripheral blood (PB) serums of 1730 breast cancer patients who were diagnosed as breast cancer in our institution from 1999 to 2012. As 30.8 pg/ml was the lowest concentration value of blood sample except zero and undetectable samples, 30.8 pg/ml was named as the positive/negative threshold. DAND5 concentration in PB serum higher than 30.8 pg/ml was defined as DAND5 positive (n=617, 35.7%), the others were DAND5 negative (n=1113, 64.3%). The clinic characteristics of the patients were shown in Table 4. There was no significant correlation between the expression of DAND5 and the clinicopathological features in the 1730 breast cancer patients which was different from the IHC dataset. We speculated that the trend of secreted DAND5 was not very coordinated with DAND5 expression in breast cancer cell. In univariate and multivariate survival analysis, positive DAND5 expression was associated with a nearly 1.58-fold and 1.4-fold increased risk of disease events occurrence (univariate: HR=1.58; 95% CI: 1.206-2.070; P=0.001; multivariate: HR=1.4; 95% CI: 1.003-1.954; P=0.048, Table 5). Furthermore, in the 1730 patients group, there were 63 patients who developed lung metastasis. Only 3 patients displayed secreted DAND5 negative. All the other 60 patients were turned to be secreted DAND5 positive. In the Kaplan-Meier analysis, except OS (P=0.523; Figure 3C), DAND5 positively correlated with poor DFS (P=0.001; Figure 3A) and DDFS (P=0.002; Figure 3B), revealing a possibility that DAND5 expression was linked to poor prognosis in BC.

**Table 4:** Relationship between DAND5 expression in peripheral blood serums and clinicopathological features in 1730 breast cancer patients for ELISA detection

Characteristics	DAND5		Number of patients (%)	P value
	Low n (%)	High n (%)		
**Total**	**1113(64.3)**	**617(35.7)**	**1730**	
Age				0.583
<50	1011(58.4)	517(29.9)	158 (88.3)	
≥50	93(5.4)	47(2.7)	140(8.1)	
Menopausal status				0.191
Pre	542(31.3)	283(16.4)	825(47.6)	
Post	562(32.5)	335(19.4)	897(1.9)	
Tumor size (cm)				0.305
2cm	559(32.3)	283(16.4)	842(48.7)	
>2,5cm	369(21.3)	235(14.6)	604(35.9)	
>5cm	32(1.8)	20(1.2)	52(3)	
Node status				0.196
Negative	698(40.3)	362(20.9)	1060(61.2)	
Positive	407(23.5)	256(14.8)	663(38.3)	
ER status				0.463
Negative	320(18.5)	168(9.7)	488(28.2)	
Positive	718(41.5)	412(23.8)	1130(65.3)	
HER-2 status				0.164
Negative	834(48.2)	487(28.2)	1321(76.4)	
Positive	71(4.1)	30(1.7)	101(5.8)	
Differentiation				0.130
I	20(1.2)	8(0.5)	28(1.7)	
II	500(28.9)	305(17.6)	805(46.5)	
III	280(16.2)	135(7.8)	415(24)	
TNM stage				0.091
I	390(22.5)	184(10.6)	574(33.1)	
II	407(23.5)	239(13.8)	646(47.3)	
III	174(10.1)	117(6.8)	291(16.9)	

Abbreviations: ER, estrogen receptor; HER-2, human epidermal growth factor receptor 2 a. Based onPearson χ2 test except for surgery type and radiation therapy, for which P is based on Fisher's exact test.

**Table 5:** Univariate and multivariate analysis for disease free survival in 1730 cases

	Univariate analysis		Multivariate analysis	
	HR (95% CI)	p value	HR (95% CI)	p value
Age	0.661 (0.206-2.117)	0.485	1.106 (0.593-2.062)	0.751
Menopausal status	0.996 (0.592-1.676)	0.989	0.800 (0.568-1.128)	0.203
Tumor grade	4.286 (2.313-7.942)	**<0.001**	4.462 (2.097-10.278)	**<0.001**
Tumor size	3.317 (2.242-4.907)	**<0.001**	1.613 (1.159-2.245)	**0.004**
Lymph node status	2.847 (2.276-3.563)	**<0.001**	2.135 (1.552-2.938)	**<0.001**
ER status	0.399 (0.236-0.674)	**0.001**	0.861 (0.596-1.242)	**0.423**
HER-2/neu status	2.304 (0.905-5.867)	0.08	1.122 (0.584-2.153)	0.73
DAND5	1.58 (1.206-2.070)	**0.001**	1.4 (1.003-1.954)	**0.048**

Abbreviations: HR, hazard ratio; CI, confidence interval; ER, estrogen receptor; HER-2, human epidermal growth factor receptor 2; a.p is based on the Cox regression test.

## DISCUSSION

DAND5, directly binding to BMP proteins functions as an antagonist of BMP. It has been reported that DAND5 is involved in regulating organogenesis, body patterning, and tissue differentiation [15-17]. One recent report revealed that DAND5 was involved in the metastasis in breast cancer [18]. In the present study, we found that the prognosis of breast cancer patients was closely related with DAND5 expression not only in the cancer tissues but also in the peripheral blood (PB) serums. DAND5 could serve as a predict serum maker in breast cancer.

As we know, many steps are critical for the formation of metastasis. Growth of neoplastic cells must be progressive, with nutrients for the expanding tumor mass initially supplied by simple diffusion. Extensive vascularization must occur and the synthesis and secretion of angiogenic factors should establish a capillary network from the surrounding host tissue. The cells can then invade blood vessels, enter the circulation and produce additional metastasis [13, 19, 20]. Therefore, the microenvironment is very important for the BC progression [21-23]. One of the factors which could influence the microenvironment is the secreted factor family [24, 25]. Cancer secreted factors can change the cancer microenvironment to promote metastasis [26, 27]. Therefore, secreted factors which were related to the recurrence and progression of diseases could be helpful biomarkers for the diagnosis and prediction.

Till now, many new secreted factors have been discovered and used for cancer predictions. A panel of miRNAs combined with CA19-9 is established to be serum biomarker in pancreatic cancer [28]. Serum Enterolactone is reported to be related with the prognosis of postmenopausal breast cancer [29]. Tumor-induced miRNA16 and miRNA378 secretion changes as regulators and biomarkers of osteolytic bone metastasis [30]. Besides, serum DKK1 is found to serve as a novel protein biomarker for the diagnosis of hepatocellular carcinoma [31].

Our group is always interested in identifying novel serum biomarkers of breast cancer. When Gao and colleagues found that DAND5, a secreted factor induced dormant breast cancer cells to undergo reactivation in the lung through a gain-of-function cDNA screen, we was highly inspired. We first validated the DAND5 function using our institute tissue array including 250 breast cancer patients. It showed that DAND5 expression in the cancer tissue directly associated with recurrent diseases of BC patients.

We then examined the function of secreted DAND5 in the microenvironment. When DAND5 was stably expressed in the breast cancer cells, the concentration of DAND5 in the culture medium of these cells significantly increased. The secreted DAND5 increased the number of pseudocapillaries of HUVEC cells in terms of completed circles, while shDAND5 decreased the numbers. It suggested that secreted DAND5 could promote pseudocapillary formation. In the xenograft mice model, DAND5 overexpression also promoted the micro-vascular formation in the xenograft tumors. Additionally, the tumors in the DAND5 group were all bigger than the control group. These results suggest that the secreted DAND5 regulated the cancer progression through changing the microenvironment.

To test our hypothesis, the validation model which contained 1730 breast cancer patients' peripheral blood (PB) serums was established. The serum concentration of DAND5 was detected through ELISA assay. In healthy control group, DAND5 concentration was extremely low and undetectable. In univariate and multivariate survival analysis, positive DAND5 expression was associated with a nearly 1.58-fold and 1.4-fold increased risk of disease events occurrence. Using the Kaplan-Meier analysis, we found that DAND5 positivity correlated with poor DFS and DDFS in breast cancer.

Taken together, we speculate that when DAND5 gene was amplified or DAND5 protein was overexpressed in breast cancer, DAND5 could be secreted from the cells into the surrounding and then affect the microenvironment to facility the cancer cell for progression. DAND5 could serve as an easily detectable serum biomarker for early prognosis and to predict the survival of breast cancer patients.

## MATERIALS AND METHODS

### Patients

Informed consent forms were signed by each participant, and appropriate ethical committee approval was obtained. For the tissue microarray, 250 primary BC tissue samples from female invasive ductal carcinoma patients (no co-morbidities reported) were randomly collected at the Department of Breast Surgery of the Fudan University Shanghai Cancer Center (FDUSCC, Shanghai, P.R. China) between 2002 and 2006. The median follow-up time was 96 months (84-141 months). All patients were female and had a median age of 53 years at the time of diagnosis. Prior to constructing the tissue microarray (TMA), each paraffin-embedded tumor sample was defined and the tumor regions marked based on H&E staining. The TMA sections were generated by the Department of Pathology at the Fudan University Shanghai Cancer Center. Briefly, tissue cylinders with diameters of 10 mm were punched from the above regions and transferred to recipient array blocks using a Tissue Micro Arrayer. The TMA included duplicate cores from different areas of the same tumor to compare the staining patterns.

For the DAND5 serum ELISA assay, 1730 blood samples were randomly collected from BC patients before primary breast surgery at the Department of Breast Surgery in FDUSCC between 1999 and 2012. The median follow-up time was 54.9 months (13-159 months). All patients were female and had a median age of 51 years at the time of diagnosis. Serum samples were aliquoted and stored at −80°C until DAND5 was analyzed via sandwich ELISA as described below.

### Immunohistochemistry

Expression levels of DAND5 (TA503555, ORIGENE) in postoperative paraffin-embedded tumor specimens from breast cancer patients and mice tumor tissues were detected with IHC. The concentrations of antibodies used are as follows: DAND5, 1:100. The Envision and diaminobenzidine (DAB) Color Kit was purchased from Gene Tech Company Limited (Shanghai, China). The staining procedures strictly followed the supplier's recommendation. The staining index (SI, range 0–9) was considered as the product of the intensity score (0, no staining; 1+, faint/equivocal; 2+, moderate; 3+, strong) and the distribution score (0, no staining; 1+, staining of <10 % of cells; 2+, between 10 % and 50 % of cells; and 3+, >50 % of cells). For DAND5 protein in this study, a moderate/strong staining (SI = 3–9) was defined as positive or high staining, and a weak or negative staining (SI = 0–2) was defined as negative or low staining.

### ELISA

A Human DAND5 Duo Set kit for ELISA (R&D Systems, Inc., USA) was used to perform serum DAND5 ELISA assays according to the manufacturer's protocol. Briefly, the plates were coated with the DAND5 capture antibody in PBS. After incubation overnight at 4°C, the plates were then washed and blocked with washing buffer. Subsequently, 10 μl of standard preparations of different densities, as well as patient blood serum samples, were added to the wells, and the plate was incubated overnight at 4°C. After the plate was washed, a 100-μl solution containing the detection antibody (300 μg/ml) was added and incubated at room temperature for 2 hrs. The wells were washed again and then incubated with a 100-μl solution containing streptavidin-conjugated horseradish peroxidase at room temperature for 20 min. Following multiple washes, the wells were treated with 100 μl of substrate solution and incubated in the dark at room temperature for 20 min. After the addition of 50 μl of stop solution to each well, the absorbance was detected at 450 nm. The online tool “Four Parameter Logistic Fit” was used to calculate the absolute concentration of DAND5 in the blood serum samples.

### *In vitro* angiogenesis model

Human Umbilical Vein Endothelial Cells (HUVEC) were suspended in culture medium from stable cell lines and then plated onto a thin layer (300 ml) of basement membrane matrix (Matrigel; BD Biosciences) in 24-well plates at 1×10^4^ cells/well. After 12 h, the medium was removed, cells were fixed, and images of cells were obtained with a light microscope (Leica) at ×20 magnification. Quantification of the tubular structures was performed by counting the number of complete circles produced by interlinking tubular HUVECs [15].

### Tumorigenicity assays and blood vessel assessment in athymic mice

Female athymic BALB/c nu/nu mice, 4–6 weeks old, were obtained from the Shanghai Institute of Materia Medica, Chinese Academy of Sciences. All studies on mice were conducted in accordance with the National Institute of Health (NIH) ‘uide for the Care and Use of Laboratory Animals'. The study protocol was approved by the Shanghai Medical Experimental Animal Care Committee. Animals were divided into four groups: MDA-MB-231/pBABE and MDA-MB-231/DAND5, MDA-MB-231/scramble and MDA-MB-231/shDAND5. Each group contained 16 mice. Cells (MDA-MB-231, 1.5×10^6^) were injected into the mammary fat pad of mice. Animals were monitored every 2 days for tumor growth and general health. Animals were sacrificed and autopsied at 6 weeks after cell inoculation. To confirm the expression of the indicated proteins, sections were cut at 50 μm intervals and stained with hematoxylin and eosin (H&E) and by IHC. Microangiography for blood vessels was performed at the Beamline BL13W1, and X-ray imaging at the biomedical application station of the Shanghai Synchrotron Radiation Facility (SSRF) in China. The maximum light size of the beam was 45 mm (horizontal) × 5 mm (vertical) at the object position at 16 keV. All animals were anesthetized by intraperitoneal injection of ketamine (200 mg/kg) (Ketanest; Pfizer, Karlsruhe, Germany). The image contrast agent microfil (Flow Tech Inc., Carver, USA) was injected into the left ventricle. Serial images of tumor blood vessels in nude mice were then recorded using SSRF.

### Statistical analyses

Correlations between clinical-pathological parameters and interested markers were evaluated using contingency tables and Pearson χ2 test or Fisher's exact test. Disease-free survival and overall survival were derived from the Kaplan-Meier estimate and compared by the log-rank test. Univariate and multivariable analysis were carried out using the Cox risk proportion model. Statistics was analyzed using SPSS (version 13.0; SPSS Company). All P values are two-sided and a P value of less than 0.05 was considered significant.

## SUPPLEMENTARY FIGURES


