# Supplementary material for: The BMP inhibitor DAND5 in serum predicts poor survival in breast cancer
Source: Oncotarget. 2016 Feb 19;7(12):14951–62. doi: 10.18632/oncotarget.7498 (PMC4924764; doi:10.18632/oncotarget.7498)
Supplement: Supplementary file 1 [file oncotarget-07-14951-s001.pdf]

SUPPLEMENTARY FIGURES

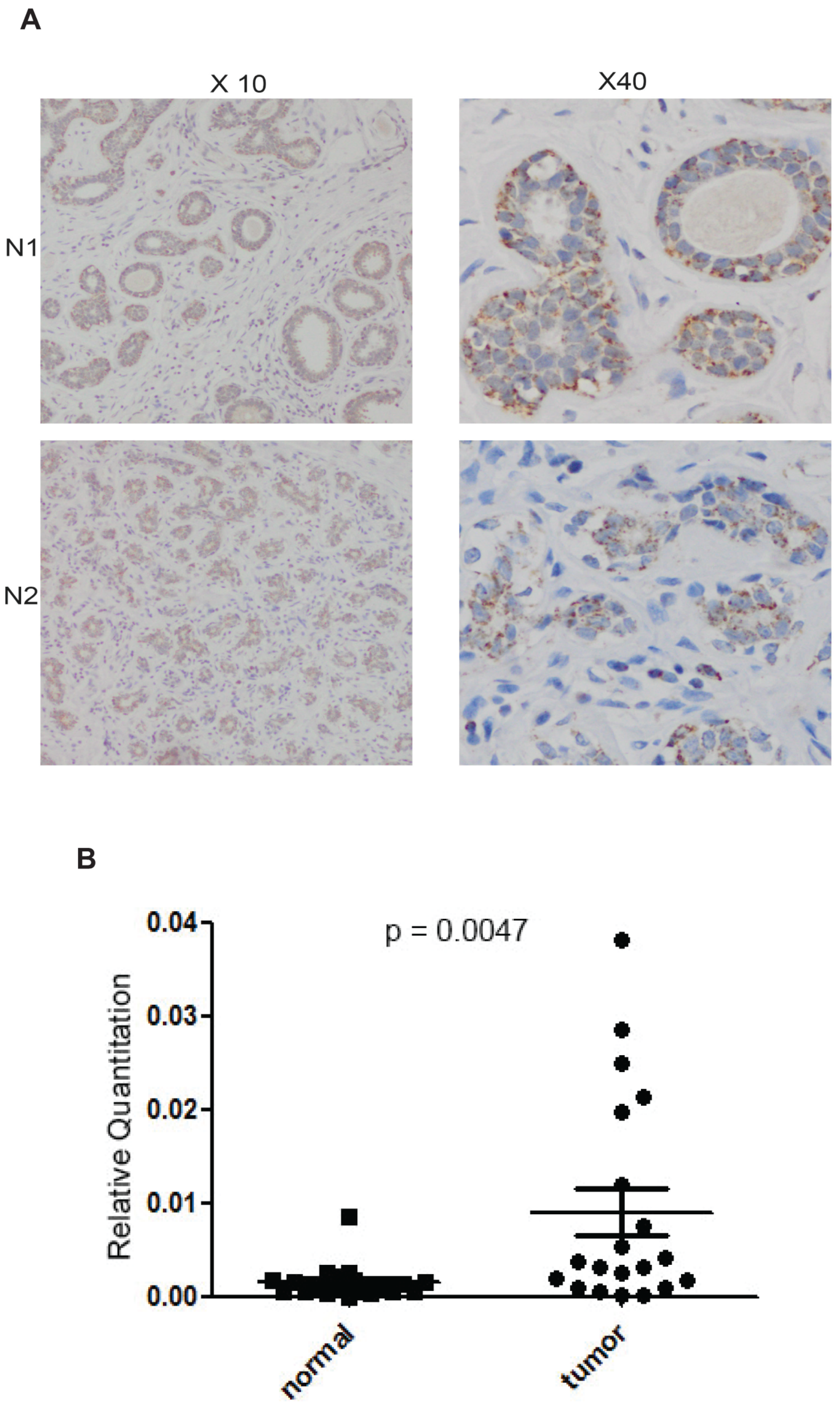

Supplementary Figure S1: DAND5 show high expression in breast cancer tissues compared with the adjacent normal breast tissues. A. DAND5 immunostaining in breast cancer and the adjacent normal breast tissues. All immunohistochemical photomicrographs are magnified 400×. **B.** Comparison of DAND5 expression levels between 20 pairs of BC tumor tissues and ANCT normal tissues by qRT-PCR analysis. GAPDH was used as an internal control.

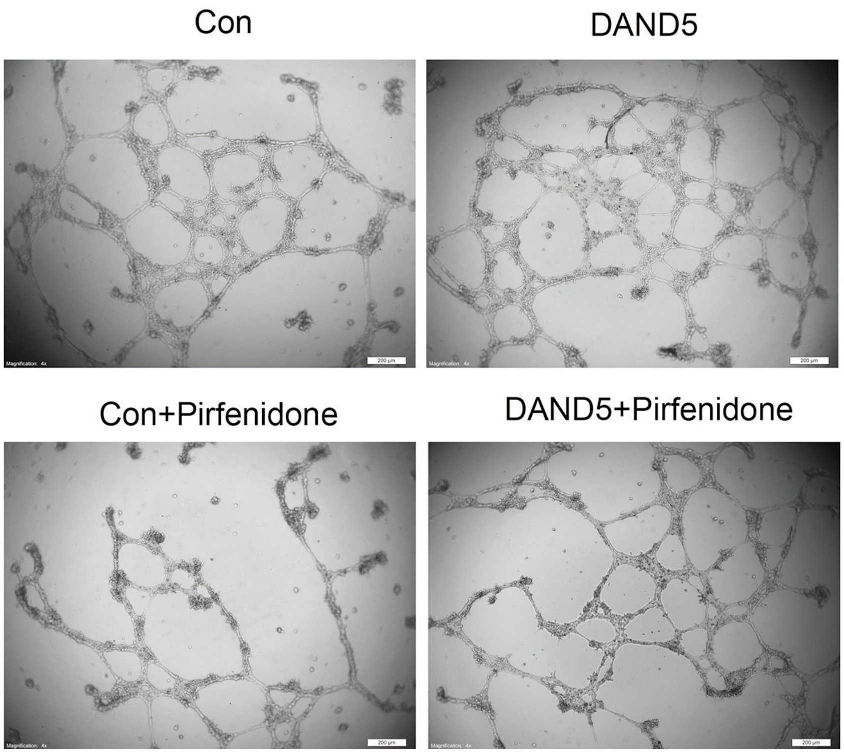

Supplementary Figure S2: TGF β blocker Pirfenidone inhibited the angiogenesis induced by DAND5. Representative pictures of pseudocapillary formation in matrigel from HUVECs in 0.1% FBS exposed to breast cancer cell culture and Pirfenidone at 12 h after cell seeding.
